# Molecular probes for cellular imaging of post-translational proteoforms

**DOI:** 10.1039/d1cb00190f

**Published:** 2022-01-04

**Authors:** Surased Suraritdechachai, Benya Lakkanasirorat, Chayasith Uttamapinant

**Affiliations:** School of Biomolecular Science and Engineering, Vidyasirimedhi Institute of Science and Technology (VISTEC) Rayong Thailand chayasith.u@vistec.ac.th

## Abstract

Specific post-translational modification (PTM) states of a protein affect its property and function; understanding their dynamics in cells would provide deep insight into diverse signaling pathways and biological processes. However, it is not trivial to visualize post-translational modifications in a protein- and site-specific manner, especially in a living-cell context. Herein, we review recent advances in the development of molecular imaging tools to detect diverse classes of post-translational proteoforms in individual cells, and their applications in studying precise roles of PTMs in regulating the function of cellular proteins.

## Abstract 2

Specific post-translational modification (PTM) states of a protein affect its property and function; understanding their dynamics in cells would provide deep insight into diverse signaling pathways and biological processes.

## Graphical abstract

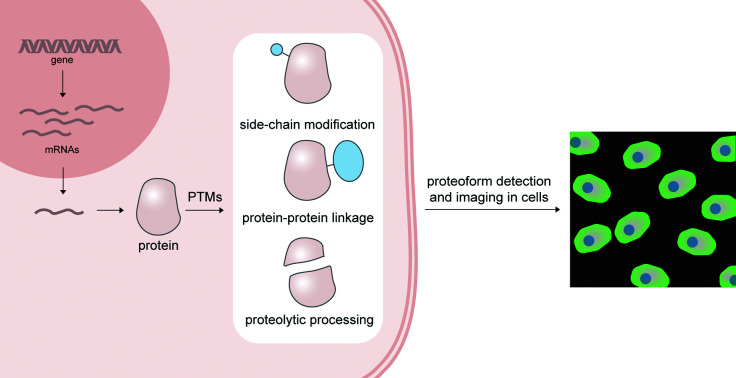

## 1. Introduction

Linking a precise protein form to its property and function in the crowded cellular context remains an outstanding challenge in biological research. While proteins can change conformations or form transient contacts with cellular macromolecules without change to their chemical compositions, they often need to be post-translationally modified to elicit function. Such post-translational modifications (PTMs) create chemically or compositionally diverse forms of a single protein—or proteoforms^1^—and are intricately controlled in space and time within the cell, diversifying the forms and functions of proteins in different contexts. In cell biological research, PTMs and proteoforms are often studied for their roles in controlling complex signaling and regulatory networks. Hundreds of PTM types are now known (and catalogued in databases like Unimod^2^) and proteins can contain multiple PTMs, creating a staggering number of heterogenous proteoforms that are only theoretically limited by protein copy numbers within the cell.^3^

Proteoforms can be discovered systems-wide *via* top-down proteomic technologies,^4^ in which intact proteoforms are analyzed in whole without digestion to peptides. Due to inherently insensitive measurements of intact protein masses and low abundance of many proteoforms in cells, systems-level detection of proteoforms may need enrichment strategies^5,6^ for specifically modified proteomes (*e.g.* for phosphoproteomes^7^ and for proteolytic proteomes^8,9^). More popular bottom-up proteomic approaches with digested peptides can also be used for proteoform detection upon coupling to appropriate peptide assignment algorithms such as correlation-based functional proteoform assessment.^10^ Subcellular information of proteoforms is often not retained in mass spectrometry-based proteomic studies unless the investigators employ cellular fractionation^11^ or proximity labeling strategies^12^ to selectively enrich proteomes from a given location within the cell. Collectively, these technologies enable global and subcellular profiling of *bona fide* proteoforms for further functional characterizations.

Technologies to synthesize proteoforms have enabled their functional characterizations *in vitro* and recently, in cells. As many post-translational modifications are catalyzed by enzymes, enzyme-mediated approaches to site-specifically install PTMs are commonly used. However, this requires prior knowledge and means of production of enzymes responsible for a particular PTM. Several PTM enzymes, including many kinases and glycosyltransferases, also modify multiple sites on the same protein, creating a heterogenous mix of proteoforms and rendering characterizations of individual proteoforms difficult. To access homogenous proteoforms, protein semisynthesis^13^ or genetic code expansion can be used. In particular, genetic code expansion can incorporate PTM-modified amino acids (phosphorylated,^14–16^ methylated,^17,18^ acetylated,^19^ ubiquitinated,^20,21^*etc.*) to any user-defined site on a given protein, allowing greater flexibility in studying site-specific functions of PTMs than protein semisynthesis. Recently, protein-specific PTM installations mediated by enzymes (*e.g.* for glycoform synthesis^22^), by genetic code expansion,^23^ by bifunctional chimera molecules,^24–26^ and by precise electrophile and oxidant delivery^27,28^ have all been accomplished in living mammalian cells, allowing roles of protein- or site-specific PTMs in regulating protein function and signaling to be established.

While proteoform synthesis methods are immensely useful, they artificially introduce PTMs onto proteins and cannot be used to study native (and often reversible) formation and regulation of proteoforms. Cell-based imaging methods to probe the formation and dynamics of post-translational proteoforms are therefore needed and would provide insight unavailable to *ex cellulo* systems and biochemical characterizations. For example, DNA damage triggers complex interactions of PTMs (including phosphorylation, acetylation, methylation, ubiquitination, and SUMOylation) on histones and other chromatin-associated proteins, resulting in the proteins’ degradation and trafficking to and from the DNA damage sites in the cell.^29^ Obtaining the spatial and temporal information of proteoforms is crucial to understanding their cellular properties (*e.g.* stability, translocation, interaction, catalytic activity), and ultimately, how they function in coordinating DNA damage responses.

PTM dynamics can be tracked in real time *via* monitoring the subcellular activity of post-translational modifying enzymes (kinases/phosphatases, methyltransferases, and glycosyltransferases) using a suite of Förster resonance energy transfer (FRET)-based biosensors containing surrogate substrates for the enzymes; efforts to create this class of technologies to probe diverse biological systems have been reviewed.^30^ However, complementary molecular tools which enable direct visualization of PTM dynamics on desired protein targets are not as well-developed. While proteins can be easily genetically tagged for visualization, most PTMs are non-genetically encoded chemical modifiers which cannot be genetically tagged. Visualizing PTM placements on specific proteins therefore often require hybrid (bio)chemical-genetic and other innovative approaches to create the desired labeling specificity.

**Fig. 1:**
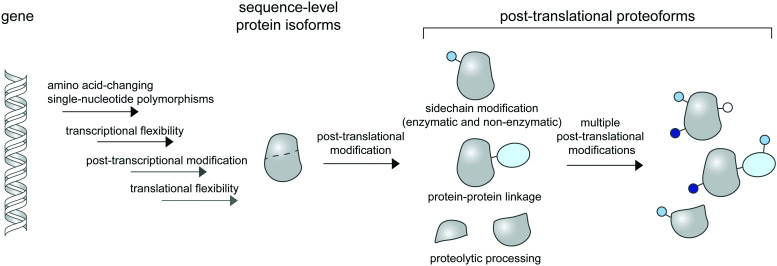
Post-translational proteoforms. Multiple sources of proteoforms exist: a gene can have single-nucleotide polymorphisms (SNPs) in its coding region; it can give rise to multiple RNA isoforms through splicing; and each mRNA form may produce multiple protein sequences through non-canonical translation initiation and termination. Thereafter, each protein sequence can be post-translationally modified—through addition of diverse chemical groups to its amino acid side chains (top), linkage to other proteins (middle), or proteolytic cleavage (bottom)—to generate post-translational proteoforms. Multiple modifications of a base protein sequence create a complex landscape of post-translational proteoforms in cells.

In this review, we discuss the available molecular tools for cellular imaging of post-translational proteoforms. We classify post-translational proteoform labeling methods based on different chemical nature of the modifications: addition of small chemical groups to protein side chains; protein–protein linkage creation; and protein cleavage (Fig. 1). Methods to visualize proteins modified with chemical groups are the most diverse; we further categorize them into technologies which provide site-specific information *vs.* those which indicate PTMs on proteins based on proximity-induced signals. As many chemical modifications on proteins are far from simple—glycosylation and ubiquitination in particular are highly complex in their structure and composition—we examine available technologies to partly address this complexity. In addition, we reviewed limited tools available for the detection of proteoforms containing non-enzymatic PTMs. Since all current probes for proteoform detection have significant—and different—limitations, we address these limitations and suggest a guideline for experimental validations of the probes when applied to proteins/systems of interest. Lastly, we discuss platforms for high-throughput and multiplexed protein imaging, which can be readily applied to imaging of cellular proteoforms, once suitable molecular probes for proteoform detection are in place.

## 2. Molecular tools to detect proteoforms involving addition of functional groups

### 2.1 Site- and PTM-specific antibodies and binders

**Fig. 2:**
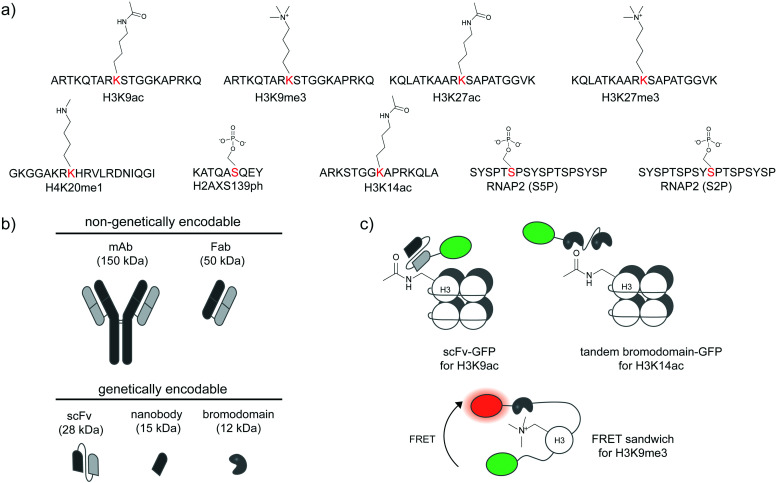
Detection of post-translational proteoforms by site-specific antibodies and binding proteins. (a) Examples of post-translational epitopes detectable by proteoform-specific antibodies and binders. Anti-H3K9ac from ref. 34; anti-H3K9me3 from ref. 34; anti-H3K27ac from ref. 34; anti-H3K27me3 from ref. 34; anti-H4K20me1 from ref. 32; anti-H2AXS139ph from ref. 39; anti-H3K14ac from ref. 40; anti-RNAP2 (S5P); anti-RNAP2 (S2P) from ref. 37. (b) Comparison of size and genetic targeting properties of antibody- and affinity-based reagents. (c) Genetically encoded detection of proteoforms by single-chain variable fragments (scFv), bromodomains, and FRET sandwiches. Proteoform-targeting moieties such as a scFv targeting H3K9ac^41^ and a bromodomain targeting H3K14ac^40^ are fused to reporters, such as green fluorescent protein (GFP). FRET sandwiches contain a fluorescent protein FRET pair and a PTM reader domain.^42^

Specific antibodies for defined PTM states of a given protein can be developed (Fig. 2a),^31,32^ most commonly through animal immunization with a synthetic peptide segment from the protein target modified with the desired site-specific PTM. Beyond immunization, directed evolution techniques that select for binders with high affinity can be used to create antibodies and binders with desired specificity. If succeeded, such reagents provide the most direct route to the detection of post-translational proteoforms in cells. A large number of site-specific antibodies for phosphorylation, methylation, and acetylation are now commercially available. Alternative proteoform-specific binders with smaller size than traditional antibodies have also been developed (Fig. 2b). For instance, the Kimura group developed FabLEM (Fab-based live endogenous modification labeling) to visualize histone modification.^33,34^ Compared to monoclonal immunoglobulin G (IgG, ∼150 kDa), Fab fragments are smaller (∼50 kDa), diffuse faster and possess capability to pass through the nuclear membranes while retaining the recognition specificity. The FabLEM method is applicable to detection of several histone modifications^34–36^ including methylation, acetylation, and phosphorylation. It has also been used to monitor phosphorylation of RNA polymerase II (RNAP2) under different polymerase activity states in cultured cells.^37,38^ Histone and RNAP2 proteoforms could be monitored simultaneously: after activation of the glucocorticoid receptor transcription factor, a decrease in histone acetylation was observed followed by an increase in RNAP2 phosphorylation levels, indicating progression to transcription elongation. In conjunction with MS2-based nascent mRNA tagging, the Fab fragment could be used to monitor specific phosphorylation states of RNAP2 as it transcribes a single gene.^38^

As direct loading of Fab fragments cannot provide sustained amount of Fab after rounds of cell division, thereby limiting long-term monitoring of proteoform states,^33^ the Kimura group further developed a genetically encodable fusion of a single-chain variable fragment (scFv, 28 kDa) and green fluorescent protein (GFP) (Fig. 2b), called modification-specific intracellular antibody or mintbody. In the first report, a mintbody for H3K9 acetylation allowed monitoring of increased acetylation levels in response to a histone deacetylase inhibitor (Fig. 2c).^41^ For *in vivo* applications, the H3K9 acetylation mintbody could monitor changes in acetylation levels during embryogenesis of transgenic Drosophila and zebrafish embryo,^41^ and also during Xenopus tail regeneration.^43^ Mintbody for H4K20me1 detection has also been developed to specifically monitor histone monomethylation in several organisms including S*chizosaccharomyces pombe* yeast, mammalian cell lines, and *Caenorhabditis elegans.*^44,45^

Single variable heavy-chain domain of antibodies (VHH), or nanobodies, are even smaller in size (15 kDa) than Fab or scFv, but elicit highly specific antigen binding compared to conventional antibodies. Derived from monomeric immunoglobulins found in camel and shark species,^46^ nanobodies can be expressed recombinantly in cells and act as intracellular antibodies similarly to scFv (Fig. 2b), but are more monomeric and stable inside cells than scFv.^47^ Various types of nanobody libraries can be prepared, and coupled to diverse selection methods—in particular to surface display technologies—to discover nanobodies with desired specificity.^48,49^ Nanobodies have been developed to detect specific proteoforms.^50–52^ A recent example is a phage display-derived nanobody specific for human H2AX histone phosphorylated at position Ser139, or γ-H2AX.^39^ A bivalent variant of this nanobody can be expressed recombinantly as a fluorescent protein fusion in a human lung carcinoma H1299 cell line, and used to visualize the formation of γ-H2AX foci in the cell nucleus, in response to genotoxic drug treatment and the resulting DNA replication stress.

Beyond antibody-based detection, several protein domains that bind histone PTMs with sequence specificity have been identified. For instance, a bromodomain can bind specifically to specific acetylated lysines on histones in live cells.^53^ A GFP-fused bromodomain can be used to visualize endogenous histone acetylation (Fig. 2c).^40^ The fluorescence signal from the bromodomain-based sensor colocalized with the H3K14ac antibody and increased in response to histone deacetylase inhibition. The sensor for histone H3K9me3 based on a chromodomain was also reported.^54^ These sensors could be coupled to a DNA-binding zinc finger domain to monitor locus-specific H3K9me3 modification,^55^ or to other histone reader domains to simultaneously report multiple histone modifications,^56,57^ or with a methyl binding domain to simultaneously visualize 5-methylcytosine.^57^

Further strategies to visualize histone proteoforms involve creation of FRET-based sandwich sensors through strategic fusion of the PTM recognition domain and the histone protein in between a fluorescent protein FRET pair (Fig. 2c). When the fused histone protein has high PTM levels, the histone reader domain folds to bind the modified histone, resulting in a change in FRET efficiency.^42,58–61^ This approach is applicable to live-cell monitoring of histone modifications including methylation, acetylation, and phosphorylation. A significant caveat is regulation of PTMs on histones embedded in these complex FRET sandwich sensors may differ from that of PTMs on endogenous histones.^40^

Beyond histone proteoforms, antibody-based reagents can be used to detect phosphorylated tau protein. Highly specific scFv for tau phosphorylated at Thr231 was evolved using yeast surface display, and has enabled phosphotau labeling in fixed cells and human brain tissues.^62^

### 2.2 Limitations of site-specific antibodies and binders

The development of antibody-based reagents is not trivial, can be costly and time-consuming, and is generally applicable to only a subset of PTMs with small, well-defined, homogeneous chemical structures (*e.g.* a phosphate group, a methyl group, or an acetyl group) which could act as a composite epitope along with the protein sequences surrounding the PTM for antibody recognition. Many small PTMs—for example the multiple states of non-enzymatic cysteine oxidation—cannot be easily recapitulated on synthetic peptides, making epitope creation for antibody selection difficult. As there is a limit to the epitope size recognizable by antibody-based reagents (typically 4–12 amino acids for protein sequences/patches^63^), developing site-specific antibody-based reagents for large PTM structures is even more strenuous; to date we have only seen few examples of site-specific antibodies for lipidated proteins (for palmitoylated PSD-95^64^) and none for glycosylated proteins.

Proteoform-specific antibodies and Fab fragments can be used for fixed-cell immunofluorescence but are seldom applicable to routine live-cell imaging due to their large size and cell impermeability. Such live-cell imaging studies to visualize post-translationally modified histones and transcription factors^34,36,37,65^ necessitate the use of invasive antibody delivery approaches such as microinjection, bead loading or electroporation.

The varying quality of commercial antibody-based reagents or their in-house development necessitate rigorous validation that the reagents can detect proteoforms with high specificity. While clear co-localization of signals from the immunostained proteoform with the signal from its parent protein is a minimal specificity threshold, we advocate for further validations using orthogonal approaches such as: genetic knockdowns of the target protein or the PTM-generating enzyme; mutagenesis of the PTM site on the protein; or chemical treatments known to modulate PTM levels on proteins of interest. For example, trichostatin A, a known histone deacetylase inhibitor, could be used to globally raise histone acetylation levels. Moreover, while intracellular expression of scFv, nanobodies, and protein domains has clear utility in monitoring specific proteoforms in live cells, one needs to finely tune the expression level of the reporter to reduce background from its off-target fraction.^40^

### 2.3 Proximity-mediated detection of proteoforms

**Fig. 3:**
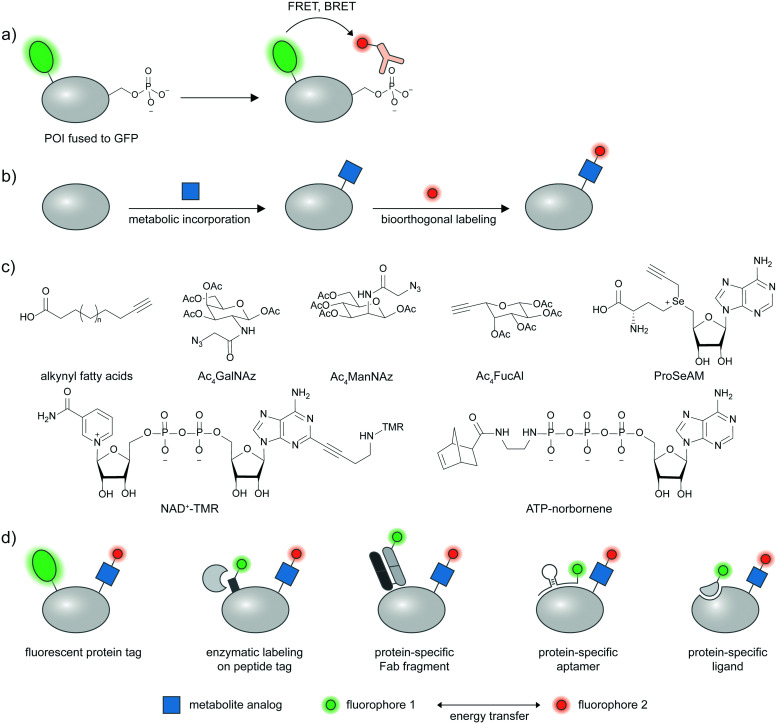
FRET based-proteoform detection *via* PTM-specific antibodies and metabolic labeling. (a) FRET-based detection of proteoforms *via* PTM-specific antibodies and a protein marker. Pan Antibodies for PTMs such as phosphoserine and phosphotyrosine are available, and can be coupled to a protein marker such as GFP and protein-specific antibodies for proteoform detection *via* proximity-induced Förster or bioluminescence resonance energy transfer (FRET or BRET). (b) General scheme for metabolic labeling on proteins. A clickable PTM substrate analog (blue square) is metabolically incorporated into cellular proteins *via* endogenous metabolic pathways. A click handle on PTM-bearing proteins is then chemically derivatized with a probe of interest (red circle) *via* bioorthogonal chemistry. (c) Examples of metabolic probes used for proteoform detection. Ac_4_GalNAz: tetraacetylated *N*-azidoacetyl galactosamine. Ac_4_ManNAz: tetraacetylated *N*-azidoacetyl mannosamine. Ac_4_FucAI: tetraacetylated 6-alkynyl fucose. ProSeAM: propargylic *Se*-adenosyl-l-selenomethionine. NAD^+^–TMR: nicotinamide adenine dinucleotide–tetramethylrhodamine conjugate. (d) Different strategies for specific protein tagging can be coupled to metabolic labeling to produce proteoform detection: fluorescent protein tagging; enzyme-mediated labeling of peptide tags; protein-specific Fab fragments; protein-specific aptamers; or protein-specific ligands.

In most cases, an antibody or binding protein for site-specific post-translational proteoforms is not available. The more generalizable approach to detect post-translational proteoforms thus is to use dual labeling: one for the protein target, and the other for the desired PTM. The proximity of the two signals is then used as an indicator of a PTM on the specific protein. For proximity-based detection of proteoforms, researchers must make three levels of choices: a protein labeling strategy; a PTM labeling strategy; and a mechanism to indicate the PTM is proximal (intramolecular) to the target protein. Here, we discuss the characteristics of the two most common proximity-induced strategies—Förster resonance energy transfer (FRET) and proximity ligation assay (PLA) in inferring PTMs on proteins, and how the choice of proximity-induced strategies may affect PTM labeling strategies. We do not discuss protein labeling in detail but provide examples of diverse strategies throughout the section; particularly, different strategies to detect proteins of interest in combination with metabolic labeling are summarized in Fig. 3.

### 2.4 FRET-based proteoform detection

FRET and PLA are most commonly employed to generate proximity-induced signals for proteoform detection. In FRET, the dipole-mediated resonance energy transfer between a donor and an acceptor fluorophore has strong distance dependence (*r*^−6^), enabling the efficiency of transfer to indicate the distance between—in our case—the label on the protein and the label on the PTM. The average diameter of proteins is ∼5 nm,^66^ acting as the upper limit of the PTM-protein label distance. Most FRET pairs have Förster radii (*R*_0_) of 2–10 nm, with a few specialized FRET pairs with even smaller *R*_0_ (down to 0.6–1 nm,^67,68^ though these have not been used in cell-based FRET assays). With suitable FRET pairs and protein/PTM labeling strategies that do not add much “bulk”, FRET should provide intra-protein resolution needed to infer the presence of a proteoform (*i.e.* the PTM and the protein are intramolecular). To complement energy transfer measurements, fluorescence correlation microscopy (FCS) can be used to assess correlated movement of the protein and PTM signals in live cells and provide further evidence of PTM–protein intramolecularity.

### 2.5 FRET-based proteoform detection with PTM-specific antibody-based reagents

In lieu of a site-specific antibody, researchers can use an antibody broadly specific to a desired post-translation modification in combination with a protein labeling strategy, and assess the extent of energy transfer between the protein and PTM labels as a proxy for proteoform presence. Pan-specific antibodies for phosphorylated residues (Ser, Thr, Tyr), different methylated lysine states, and acetylated lysine are widely commercially available. A pan-phosphohistidine antibody was reported.^69^ Antibodies for a range of carbohydrate epitopes are available, albeit with lower affinities than traditional antibodies against proteins/peptides.^70^ Antibodies against hydrophobic modifications like lipid PTMs are notoriously difficult to generate, though a pan-palmitoylation antibody was reported.^71^

Under such a FRET-based readout (Fig. 3a), the proximity-induced FRET signal of a labeled antibody and GFP-fused proteins has allowed visualization of phosphorylation in a protein-specific manner.^72–74^ In the seminal paper by the Bastiaens group, a GFP-fused epithelial growth factor receptor (EGFR) was recombinantly expressed in MCF7 cells and a cy3-labeled Fab fragment specific for phosphotyrosine was microinjected into the cells.^72,73^ FRET occurred when EGFR was phosphorylated in response to an externally supplied EGF ligand and measured *via* the fluorescence lifetime imaging technique (FLIM)-FRET.

### 2.6 FRET-based proteoform detection with metabolic labeling

In metabolic labeling, synthetic PTM substrate analogues are modified onto proteins by endogenous metabolic pathways. A biorthogonal functional handle on the metabolite analogues—such as an alkyne or an azide—can then undergo click chemistry derivatization with a fluorophore conjugate to provide detectable signal (Fig. 3b). Various PTMs can be labeled metabolically including lipidation, acetylation, and glycosylation.^75,76^ Combining the signal from metabolic labeling with a protein identification strategy allows imaging of these PTMs in a protein-specific manner. Structures of metabolic probes from studies highlighted below are shown in Fig. 3c, and different strategies to detect proteins of interest are summarized in Fig. 3d.

A fluorescent protein fusion tag can readily be coupled to metabolic labeling (Fig. 3d), and the proximity of the metabolic tag and the protein can be read through FRET signal. For example, a glucose transporter GLUT4–EGFP fusion was metabolically labeled with Ac_4_ManNAz and showed a trans-membrane FRET signal after the Ac_4_ManNAz moiety was derivatized with a rhodamine–alkyne.^77^ This enabled live-cell monitoring of accumulation of sialylated GLUT4 after insulin introduction, and internalization of sialylated GLUT4 after insulin removal. Glycosylation of cytosolic proteins such as tau, OGT, and Akt1 could be visualized in a similar manner using FLIM-FRET.^78,79^ Investigating methylation on GFP-fused proteins of interest is also possible through the use of a propargyl *Se*-adenosyl methionine (ProSeAM) analogue (Fig. 3c) and subsequent cy3–azide conjugation.^80^ SAM-based labeling has been applied to study the subcellular localization of Foxo1 which is affected by its methylation state, *via* FLIM-FRET. Poly(ADP-ribos)ylation of ARTD protein was also reported using EGFP fusion and a fluorophore conjugated NAD^+^ analog (Fig. 3c).^81^

To avoid interference to protein function from the large GFP tag, a strategy that uses a smaller tag such as LplA acceptor peptide (LAP) and enzymatic labeling has been developed (Fig. 3d).^82,83^ The LAP tag is recognized by lipoic acid ligase (LplA) mutants capable of covalently linking diverse small-molecule probes onto the tag. Using dual labeling with a sugar analogue Ac_4_ManNAl and an azide-containing LplA substrate, and subsequent bioorthogonal conjugation of a FRET donor and acceptor pair, sialylation of integrin αXβ2, EGFR, and growth factor-beta receptor type I (TβRI) could be specifically imaged in live cells. The authors highlighted the advantage of the smaller LAP tag in placing the fluorophore closer to the labeled glycan moiety in comparison to an EGFP fusion, resulting in higher FRET efficiency.

Live cell-compatible endogenous protein labeling techniques can also be used in combination with metabolic labeling to investigate proteoforms (Fig. 3d). An external Fab fragment conjugated to a fluorophore was used to visualize cell–surface integrin αVβ3. Metabolic labelling with Ac_4_ManNAz and subsequent derivatization with a cyclooctyne probe allows specific imaging of the sialylated protein *via* FRET.^84^ Exploiting native protein–protein interactions, labeled interleukin protein was used to tag a FRET donor to a surface protein interleukin 36 receptor while a FRET acceptor was installed *via* metabolic labeling with Ac_4_ManNAz (Fig. 3d).^85^

Through Systematic Evolution of Ligands by Exponential Enrichment (SELEX), nucleic acid aptamers can be evolved to bind a protein target with high affinity and specificity (Fig. 3d).^86,87^ Protein tyrosine kinase 7 (PTK7), a cell–surface protein overexpressed in subtypes of leukemia, could be recognized by an aptamer hybridized with a cy3-labeled oligonucleotide. The sialylation was probed by Ac_4_ManNAz and click derivatization with cy5–alkyne,^88^ enabling the resulting cy3–cy5 FRET signal to indicate sialylated PTK7. The FRET signal between cy3 and cy5 could be further enhanced through metal enhanced fluorescence, using a silver nanoparticle-functionalized aptamer.^89^

Expansion of metabolic probes for more types of PTMs would further enrich proteoform studies. In a recent example, a cell-permeable ATP analog bearing a norbornene group at the gamma-position was reported as a novel metabolic labeling tool for phosphorylation (Fig. 3c).^90^ The transfer of the phospho-norbornene group by purified or cellular kinases (the latter demonstrated *via* cell lysates) was confirmed *in vitro* on a peptide substrate, and *in cellulo* on murine double minute protein (MDM)–EGFP fusion. The norbornene appendage can then be chemically derivatized with tetrazine–probe conjugates *e.g.* tetrazine–cy3. The FRET signal between EGFP and cy3 informs phosphorylation extent on MDM and can be used to assess effects of phosphatase inhibitors on MDM phosphorylation.

### 2.7 Limitations of metabolic labeling

The major caveat with metabolic probes, especially ones with significantly altered or bulky motifs, is their potential interference with the native function of the PTM, and of the protein being modified; functional assays are therefore needed to validate the use of these analogs. For instance, some fluorescently labeled ATP analogs are known to have reduced activity with ATP-processing enzymes.^91^ The ProSeAM SAM analog was carefully characterized and found to be accepted by only a subset of lysine methyltransferases, despite its small propargyl modification.^92–94^ Beyond effects on PTM transfer processes, functional effects on the receiving end—the proteoform—must be carefully characterized. Continual developments to create minimally perturbative tools to label PTMs (for instance, the development of isosteric fluorinated cofactors taggable by the fluorine-thiol displacement reaction^95^) are crucial to the characterization of native proteoform function.

Metabolic probes can perturb the physiological relevance of intended studies through disruption of metabolic balance and gross toxicity to cells;^96^ researchers should keep in mind to assess cellular toxicity or gross effects of the metabolic probe and titrate its dose accordingly. Some metabolite analogs such as ProSeAM and the NAD^+^ analog are not membrane-permeable and must be loaded invasively *via* liposomes or electroporation.^80,81^ Efforts to engineer biosynthetic pathways to create cell-impermeant metabolite analogs intracellularly from cell-permeant precursors are promising,^97,98^ although these have not yet been applied to proteoform detection.

The orthogonality of certain metabolite analogs with respect to endogenous enzymatic processing has enabled cell-selective PTM and proteoform tagging, through cell-selective expression of engineered PTM transferase enzymes which can utilize these analogs. This bump-and-hole strategy to create orthogonal enzyme–metabolite analog pairs has been applied to methyltransferase,^92,99^ acetyltransferase,^100^ and GalNAc transferase enzymes.^101^

### 2.8 PLA-based proteoform detection

**Fig. 4:**
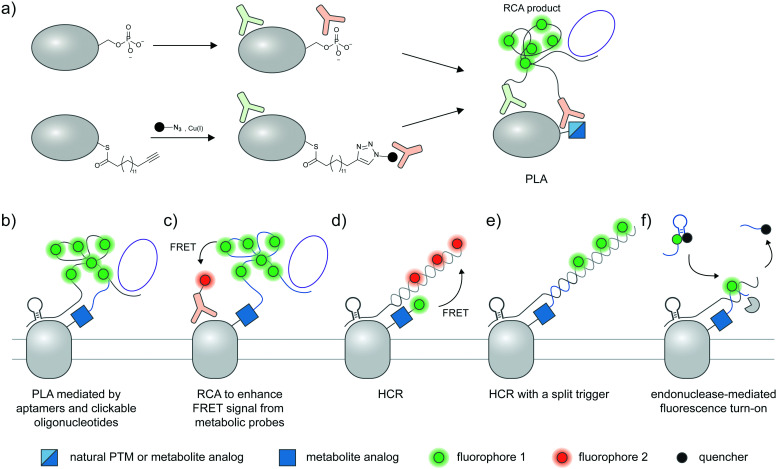
Strategies to amplify signal with nucleic acid probes for proteoform detection. (a) Proximity ligation assay (PLA) typically uses two oligonucleotide-labeled antibodies—one for the desired protein, and the other for the desired PTM—for proteoform detection. Connector oligos are used to join the oligonucleotide probes in proximity, the complex of which is then covalently linked by a DNA ligase to generate a circular DNA template for rolling circle amplification (RCA). The long single-stranded RCA product is then hybridized with fluorophore-labeled oligonucleotides. PTM antibodies for PLA can be pan-PTM antibodies (top path). Antibody recruitment to the PTM site can also be mediated by metabolic labeling and click derivatization (bottom path). (b) Protein-specific aptamers are also used to attach PLA oligonucleotides, and coupled with clickable oligonucleotides installed *via* metabolic labeling. (c) RCA can be used to specifically amplify the signal on the PTM marker, allowing more sensitive detection of the proteoform upon coupling to FRET.^106^ (d) Hybridization chain reaction (HCR) initiates assembly of labeled oligonucleotides *via* a trigger sequence at the aptamer-binding site on a specific protein. Such an extended assembly serves as an efficient FRET acceptor to a FRET donor installed *via* metabolic labeling.^107^ (e) HCR with a split trigger.^108^ Here, the trigger to initiate hybridization is split: one half is placed on the protein label (*via* an aptamer); the other half is placed on the PTM label (*via* metabolic then click labeling). Proximity-dependent reconstitution of trigger halves initiates hybridization. (f) A filter beacon architecture based on hybridization of oligonucleotides directed to a specific protein (*via* aptamers) and a PTM (*via* metabolic then click labeling).^109^ The hybridized sequence serves as a recognition site for a nicking endonuclease, resulting in cleavage and release of a fluorescence quencher and generation of fluorescence signal.

A standard proximity ligation assay (PLA) labels two targets using a pair of antibodies labeled with oligonucleotides (Fig. 4a).^102,103^ In proximity, the two tailored oligonucleotides can hybridize with a DNA template and ligated by T4 DNA ligase to form a circular DNA. This circular DNA acts as a template for rolling-circle amplification (RCA) to form a massively extended DNA product, which can be sensitively visualized by hybridization with a fluorophore-labeled oligonucleotide. PLA is versatile and has been widely used to visualize protein–protein interactions^103,104^ and post-translational proteoforms, the latter through the use of one antibody targeting the protein and the other targeting the PTM.^105^ Beyond antibody-mediated labeling, PLA can be coupled with other protein and PTM labeling strategies, provided that these strategies enable linkage of the PLA oligonucleotides to moieties of interest.

### 2.9 PLA-based proteoform detection with PTM-specific antibody-based reagents

PLA has been used to assess the phosphorylation extent of several proteins including ERK2 and SHC in K562 cells *via* a combination of the pan-phosphotyrosine antibody and several antibodies for target proteins (Fig. 4a, top).^110^ In another work, PLA with pan-phosphoserine antibody was used to study the regulation of phosphorylation of an endothelial transcription factor EGR by angiopoietin I activation of the PI3K/Akt pathway.^111^ In addition to cell lines, PLA can be performed in tissue sections to image endogenous phosphorylated proteins.^112,113^ Beyond phosphorylation, PLA has been used to monitor other protein-specific PTMs such as methylation of glucocorticoid receptor^114^ and acetylation of a transcription factor Foxp3.^115^

PLA can be further coupled to imaging flow cytometry, in a method called proximity ligation imaging cytometry (PLIC),^116^ to simultaneously sort cell populations while providing subcellular localization information of specific proteoforms. In this work, PLIC was used to assess levels of acetylation of Aire protein in mouse medullary thymic epithelial cells (mTECs), which are low-abundant cell populations in thymus tissue, using anti-Aire and anti-acetylated lysine antibodies.

### 2.10 PLA-based proteoform detection with metabolic labeling

Hannousch *et al.* reported a method to image palmitoylation of Wnt protein in mouse fibroblast L cells.^117^ Cells expressing Wnt protein were treated with 15-hexadecynoic acid (Fig. 3c), a clickable palmitoic acid analogue, then labeled with either biotin or Oregon Green to be recognized by an antibody. A palmitoylated Wnt signal was generated by proximity ligation assay from antibody targeting the tag and the protein (Fig. 4a, bottom), allowing the researchers to visualize the palmitoylated protein as it is trafficked through the secretory pathway. Later, the method was applied to study palmitoylation of sonic hedgehog, tubulin, and H-Ras in mammalian cells.^118^ PLA is compatible with tagging of various sugar analogues and has enabled detection of EGFR sialylation, EGFR fucosylation, and GalNAcylation on MUC1, a membrane protein known to be highly glycosylated.^119^

### 2.11 Limitations of PLA for proteoform detection

The reported spatial resolution of PLA is ∼40 nm, based on the approximate dimensions of two primary antibodies and the bridging oligonucleotide probes^104^ (we have not seen experimental measurements of spatial resolution provided by PLA). Such spatial resolution is simply not sufficient for intramolecularity between the PTM and the protein to be inferred. In most PLA setups for proteoform detection, the resolution is exacerbated through the use of two-tiered (primary and secondary) antibodies, degrading the spatial resolution of signal generation by further ∼30 nm. Rigorous validations are therefore needed when PLA is used for proteoform detection. Variations of PLA and other nucleic acid-based techniques with potentially improved spatial resolution are discussed in the next section.

Due to the exponential nature of nucleic acid amplification, PLA signals can suffer from saturation effects and are generally only semi-quantitative.^120^ Researchers should exercise caution when making quantitative interpretation (*e.g.* changes in PTM levels of proteins upon cell stimulation) using PLA signals. The punctate appearance of PLA signals—from individual massive RCA amplicons—reflects that only a fraction of proteoform molecules of interest is labeled; this is due to the stochastic nature of the enzymatic DNA amplification process.^120^ PLA is also limited to fixed cells due to the use of antibodies, PLA reaction conditions, and hybridization-based labeling conditions.

### 2.12 Variations of PLA for proteoform detection

Instead of bulky antibodies, oligonucleotides for PLA can be directed to protein targets *via* DNA aptamers, or to PTM targets *via* clickable metabolic probes. Since aptamers (∼2 nm)^121^ are smaller than antibodies, such combined use of smaller tags obviates the ∼30–60 nm uncertainty added by antibodies and may permit visualization of intramolecular PTM/protein relationships. In an example from the Xie group, PD-L1 is labeled with a protein-specific aptamer, metabolically labeled with Ac_4_ManNAz, and click-derivatized with an oligonucleotide probe (Fig. 4b).^122^ Proximity of the aptamer and the probe allowed ligation of additional oligonucleotides to generate circular DNA for subsequent RCA amplification. With this strategy, the researchers were able to distinguish MDA-MB-231 cells positive for glycosylated PD-L1 from PD-L1-negative BT-474 cells *via* confocal microscopy and FACS.

FRET can be used in conjunction with RCA to take advantage of the strengths of both proximity-induced techniques: FRET provides intra-protein resolution of proteoform imaging, while RCA allows multiple labeling of FRET donors/acceptors so detection sensitivity is enhanced. Here, the oligonucleotide installed on the PTM-containing protein *via* a metabolic probe was designed to hybridize with a DNA padlock probe; a ligation reaction then generated a circular DNA template for subsequent RCA and hybridization of donor fluorophore probes (Fig. 4c). Employing a protein-specific antibody conjugated to a FRET acceptor fluorophore, this approach was used to image GalNAcylation of endogenous MUC1 in MCF-7 cells and HA-tagged GPC3 in HEK293T;^106,123^ in both cases the FRET signal was increased upon performing RCA.

### 2.13 Proteoform detection with hybridization chain reaction and endonuclease-mediated filter beacon

Recently, a hybridization chain reaction (HCR) was used to probe protein-specific glycosylation on the cell surface. This technique is an enzyme-free signal amplification based on hybridization of two hairpin nucleotides that is partly complementary to each other (Fig. 4d).^124,125^ The trigger DNA hybridizes with one hairpin DNA, exposing an overhang that is complementary to another hairpin DNA. A series of hybridization events leads to the amplification of the fluorescent signal provided that one DNA hairpin is labeled with a fluorophore.

HCR was recently demonstrated by the Wang group as a method to detect sialylated PTK7. An aptamer sgc8 with a trigger sequence was designed to target PTK7. The protein can undergo metabolic labeling with Ac_4_ManNAz to install a FRET donor, followed by HCR with a hairpin DNA FRET acceptor, allowing the FRET signal from sialylated PTK7 to be detected.^107^ The same group later developed an assay to visualize sialylated PTK7 using a split trigger with one half on the aptamer probe and the other half on the clickable DNA (Fig. 4e).^108^ The hybridization of the hairpin for HCR occurs when the split triggers are in proximity. This approach helps reduce non-specific amplification and bypass the need for FRET, which generally provides low signal and dynamic range. The trigger sequence could also be protected within a hairpin structure, which can be unmasked upon hybridization with an oligonucleotide installed by metabolic labeling to allow HCR.^126^ This approach was used for fluorescence detection of sialylated proteins in cell lines, zebrafish larvae,^126^ and in a tumor model in BALB/c mice, the latter through coupling with photoacoustic imaging.^127^

In another DNA-based approach, the Ding group reported an elaborately designed clickable molecular beacon probe which could be installed *via* Ac_4_ManNAz metabolic labeling to visualize sialylation on MUC1 (Fig. 4f). Here the glycoform was detected *via* a MUC1-specific aptamer, which could hybridize with a Ac_4_ManNAz-directed clickable oligonucleotide. Hybridization generated a recognition site for a nicking endonuclease, resulting in cleavage and release of a fluorescence quencher appended to the clickable oligonucleotide.^109^ In another work, an aptamer-assisted labeling was demonstrated using an aptamer hybridized with a fluorophore-labeled clickable oligonucleotide.^128^ Such assisted labeling of a mesenchymal–epidermal transition factor (MET) metabolically labeled with Ac_4_ManNAz showed higher fluorescent signal compared to unassisted labeling.

## 3. Detection of proteins modified with ubiquitin and ubiquitin-like proteins

Covalent modifications of cellular proteins with a small, 8 kD ubiquitin (Ub) protein trigger proteasome-mediated degradation as well as other proteasome-independent cellular processes. Beyond ubiquitin, proteins can be covalently linked to other ubiquitin-like proteins such as Small Ubiquitin-like Modifier (SUMO) and Neural precursor cell Expressed, Developmentally Down-regulated 8 (NEDD8).^129^ Strategies to visualize Ub/Ub-like modifications for cellular imaging have been reviewed.^130^ In general, FRET,^131,132^ BRET^133^ or PLA-based proximal detection^134–136^ based on dual labeling of the Ub/Ub-like proteins and their protein substrates—*via* GFP tagging or immunofluorescence for both the protein target and the Ub/Ub-like modifier—can be accomplished.^135,136^

Another common strategy to visualize Ub/Ub-like modifications in living cells is to use bimolecular fluorescence complementation (BiFC) *via* split protein tagging. Split fluorescent proteins are most commonly used for cellular imaging applications, though other split protein systems^137^ can also be used for luminescence-based imaging or other modes of detection. Ubiquitin-mediated fluorescence complementation was reported in 2004.^138^ In the study, a transcription factor Jun was fused to the C-terminal fragment of yellow fluorescent protein and the complementary fragment was fused to ubiquitin. The reconstituted fluorescent signal indicated that ubiquitinated Jun was exported out of the nucleus and targeted to the lysosomes. Ubiquitination of transcription factor FOXO^139^ as well as SUMOylation of Jun and ATF6 in living cells were visualized by the same approach.^140^ A library of proteins can be screened for its SUMOylation extent by a similar split-protein approach (one fragment of the split fluorescent protein on the protein library; the other fragment on SUMO). SUMOylated proteins can be detected in a high-throughput manner by FACS, and their subcellular localization assessed by microscopy.^141^ While BiFC reporters are generally irreversible,^142^ reversible split protein reporters^143,144^ were recently developed and are better suited to monitor dynamic PTMs.

Proteins can be polyubiquitinated, and such polyubiquitination can occur on different attachment sites (*e.g.* Lys29, Lys48, Lys63). The complexity is further increased with PTMs on Ub, or incorporation of other Ub-like proteins to form a hybrid chain.^145,146^ These diverse structures of polyUb/Ub-like proteins play different roles within the cell ranging from signaling to degradation.^147^ Expression of Ub fused to fluorescent proteins has been used to visualize several forms of polyubiquitination based on proximity of two ubiquitins.^148–151^ This approach is straightforward but requires ectopic ubiquitin expression. Other approaches using antibodies and protein binding domains that bind a specific linkage form of polyubiquitination have been reported.^130,145^ Affimers targeting the Lys6 and Lys33 chains have been developed and their utilities demonstrated in western blot, imaging, and pull-down assays.^152^ A synthetic Fab fragment for the Lys29 chain was evolved from phage display, and used to enrich Lys29 chain-modified proteins for mass spectrometry-based proteomics studies.^153^ Imaging with fluorescently labeled Fab highlighted the concentration of K29 polyubiquitin signal around the midbody at telophase during the HeLa cell division. These specialized PTM detection tools could be combined with a protein identification signal to visualize protein-specific Ub/Ub-like modifications.

Beyond proteoform localization, a three-pronged detection approach to visualize a proteoform participating in protein–protein interactions was developed. The assay coupled FRET with BiFC to enable detection of protein ternary complexes,^154^ and was used to image interactions of a SUMOylated transcription factor BMAL1 with CREB-binding protein; this interaction resulted in the activation of the CLOCK-BMAL1 circadian clock.^155^

## 4. Detection of proteolytic proteoforms

Proteolytic processing can generate smaller proteoforms with distinct properties and functions from their pro-proteins. The resulting proteolytic proteoforms can act as hormones (insulin, adrenocorticotropic hormones) and signaling molecules (neuropeptides); some are associated with disease pathology (amyloid β peptides). Akin to the monitoring of protein–protein conjugation (*e.g.* ubiquitination), proteolytic proteoforms can be monitored *via* dual/multiplexed labeling of protein parts and subsequent proximity-based or simpler multicolored imaging readouts. The challenge lies in judicious epitope selection (for antibody-based detection), or placement of the recognition tag (for recombinant tag-based detection) to allow specific detection while minimizing interference with the complex, often sequential proteolytic processes.

**Fig. 5:**
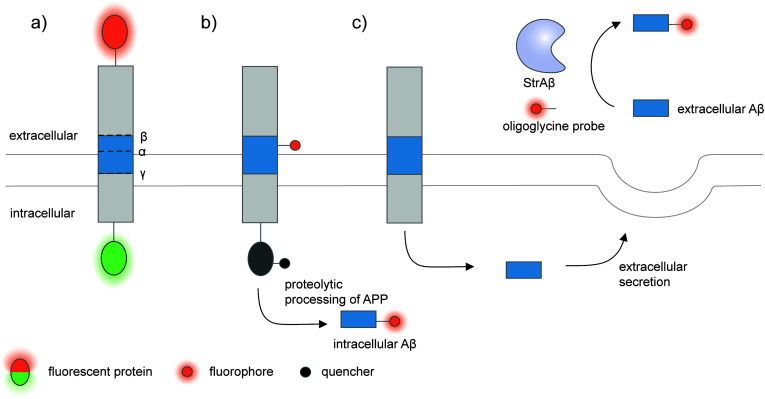
Strategies to detect proteolytic proteoforms derived from amyloid precursor protein (APP). Key proteolytic cleavage sites on transmembrane APP by α-, β-, and γ-secretases are illustrated. (a) APP can be dually labeled with two fluorescent proteins (red and green ovals), and its N- and C-terminal fragments upon cleavage separately tracked.^159,160^ (b) Genetic code expansion-mediated labeling of intracellular amyloid-β peptides.^162^ The amyloid-β-containing segment within APP is site-specifically labeled with an organic fluorophore (red circle) *via* genetic code expansion. The fluorescence signal from this label is suppressed prior to proteolysis by an intramolecular quencher (dark grey circle) appended to APP *via* enzyme-mediated labeling. Proteolysis along the endocytic pathway generates amyloid-β peptides and separates the fluorophore-labeled amyloid-β from the quencher-labeled precursor, resulting in enhanced fluorescence. (c) Sortase-mediated labeling of extracellular amyloid-β peptides.^163^ An engineered sortase specific for amyloid-β, called StrAβ, catalyzes transpeptidation to link an oligoglycine-fluorophore conjugate to the C-terminus of endogenous amyloid-β.

To monitor proteolysis of specific precursor proteins, a pair of fluorophores could be attached to the target precursor protein (Fig. 5a). For example, a proteolytic sensor of neuregulin 1 (NRG1; a membrane protein that releases its ectodomain as an intercellular signal) was developed by fusing the protein with mCherry and EGFP at the extracellular N-terminal and the intracellular C-terminal respectively.^156^ The NRG1 cleavage could be monitored in cell culture and in neurons of zebrafish embryo. The imaging and fluorescence ratio illustrated that NRG1 shedding occurred in the axon more than in the neuron cell body. Ectodomain shedding of TGFα can be monitored in a similar manner.^157^ Similarly, processing and secretion of insulin in live Min6β cells could be visualized by fusion of GFP and mCherry to the A-peptide and C-peptide of proinsulin. Changes in the GFP-to-mCherry fluorescence intensity ratio indicated release of mature insulin, allowing effects of stimulation and drugs on insulin secretion to be measured.^158^ The approach is also applicable to the study of processing of amyloid precursor protein (APP) fused with two fluorescent proteins at its two terminis.^159–161^ After β-secretase-mediated cleavage, differential subcellular sorting of the N- and C-terminal APP fragments could be tracked within the cell.^159,160^

Many proteolytic proteoforms are small peptides, which are impossible to tag with large fluorescent proteins^164^ nor specifically detected with antibodies. To enable detection of such proteolytic peptides in cells, minimally invasive labeling strategies such as genetic code expansion can be used. Genetic code expansion employs an orthogonal aminoacyl–tRNA synthetase and tRNA pair to incorporate an unnatural amino acid typically at an amber stop codon introduced to the gene of interest.^165^ Diverse functional groups could be installed and further derivatized with fluorophores and tags using bioorthogonal chemistry. To distinguish the labeling signal of the processed peptide from that of the pro-protein, two fluorophore labels—one embedded in the processed peptide segment *via* genetic code expansion, and the other on the pro-protein—are required.^166^ Alternatively, a molecular beacon labeling strategy to visualize processed peptides has been developed (Fig. 5b).^162^ Here, a fluorophore was embedded in the peptide-containing segment of the pro-protein *via* genetic code expansion, while a fluorescence quencher was attached to another site within the pro-protein. Fluorescence was suppressed *via* distance-dependent FRET-based quenching while the pro-protein was intact, but elicited when the pro-protein was proteolyzed and the processed peptide liberated. Such molecular beacon labeling was used to label ∼40-amino-acid amyloid β peptides (Aβ, labeled with cy5 *via* genetic code expansion) as they are processed from APP (labeled with QSY21 quencher *via* HaloTag) along the endocytic pathway of live cells.

While genetic code expansion allows detection of proteolytic peptides, the method requires exogenous expression of engineered protein translation machineries (particularly an orthogonal aminoacyl–tRNA synthetase/tRNA pair) and is difficult to extend to *in vivo* and clinical work. Aβ plaques are pathological hallmarks of Alzheimer's disease, and the majority of Aβ processed from APP is secreted and aggregated in the extracellular fluids of the brain.^167,168^ To enable the detection of endogenous Aβ found in clinical fluids, the Liu group used yeast display-based directed evolution to change the substrate specificity of *Staphylococcus aureus* sortase A, such that the evolved enzyme, called StrAβ, now recognizes an LMVGG sequence at the termini of endogenous Aβ as a transpeptidation motif (Fig. 5c).^163^ Diverse probes (biotin, fluorophores) linked to a triglycine can then be conjugated to Aβ *via* StrAβ. StrAβ was successfully used to modify Aβ in human clinical cerebrospinal fluid (CSF) samples, allowing not only their direct detection, but further characterizations of its aggregation kinetics and a potential way to mark Aβ for targeted degradation.

## 5. Detecting non-enzymatic proteoforms

The preceding sections discuss the detection of proteoforms originated from enzymatic post-translational modifications; these are traditionally viewed as main regulators and diversifiers of protein function. On the other hand, the importance of non-enzymatic PTMs beyond markers of cellular stress has only begun to emerge (recent reviews here^169–171^). Non-enzymatic PTMs are generated from reactions between amino acid sidechains with suitable nucleophilicity or redox activity, and reactive (often oxidative or electrophilic) metabolites. Several non-enzymatic PTMs are now known to be reversible^170^ through changes in oxidative states of the cellular microenvironment or even through enzymes, providing relevance of this class of PTMs in protein regulation. Due to their recently recognized importance, there are much fewer cellular imaging tools for non-enzymatic proteoforms; the current technological gap provides opportunities for chemical biologists to develop new detection tools for non-enzymatic proteoforms, *via* design of clickable reagents or exploitation of unique reactivities of these PTMs.

**Fig. 6:**
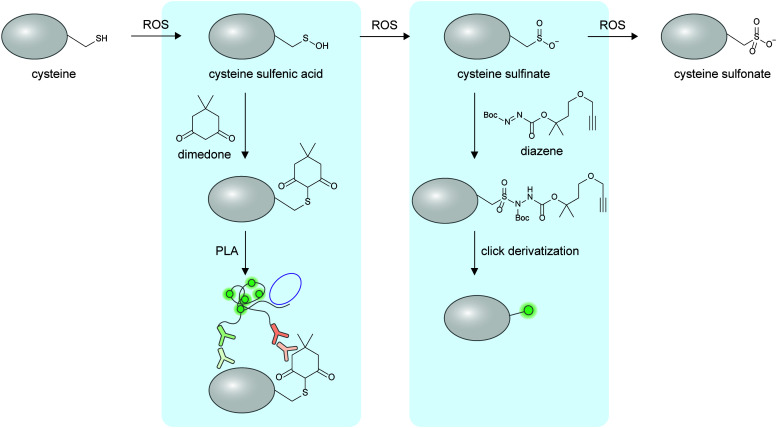
Detection of non-enzymatic oxidative proteoforms. The thiol group of cysteine can be oxidized sequentially to sulfenic acid, sulfinate, and sulfonate. Sulfenylated cysteines can be chemically derivatized with dimedone, the product of which can be detected with an antibody. Sulfinylated cysteines can be derivatized with electrophilic diazene compounds to install bioorthogonal functional groups and/or fluorophores. ROS, reactive oxygen species.

Oxidation and nitrosylation of multiple amino acid sidechains—particularly cysteine—are examples of non-enzymatic PTMs resulted from cellular oxidants such as reactive oxygen species (ROS) or reactive nitrogen species (RNS). In some cases, these oxidative PTMs are known to dynamically regulate protein function.^172^ While imaging of almost all protein-specific oxidative PTMs is currently not possible, certain oxidative PTMs can be chemically derivatized to provide a detection handle on specific proteins (Fig. 6). To detect protein-specific sulfenyl cysteine residues, cells can be treated post-fixation with dimedone, followed by immunostaining with an anti-dimedonylated cysteine antibody. A protein-specific antibody could then be used to perform PLA, and has enabled the study of the spatiotemporal regulation of oxidized SH2 protein.^173^ The higher oxidation state of cysteine—sulfinylation—can also be probed using electrophilic diazenes to form stable sulfonamide adducts.^174^ The utility of electrophilic diazene probes could likely be extended to specific Cys-sulfinylated proteoform imaging.

## 6. Monitoring proteoform turnover in cells

Proteoform labeling with genetically encoded and/or metabolic probes is generally compatible with real-time, dynamic imaging, though careful validations are needed to ensure the labels minimally interfere with the PTM installation/removal processes. FRET-based histone modification sensors are likely the most developed for real-time dynamic measurements. For example, a FRET-based reporter based on full-length H3 and a chromodomain HP1 could monitor the dynamics of H3K9me3 during the division of HeLa cells, and can be coupled to H3S10 phosphorylation monitoring *via* a second FRET sensor which detects phosphorylation on a small H3 peptide.^61^ In another work, dynamics of monomethylation of endogenous H3K20 during the cell cycle was tracked in real time by a specific mintbody.^44^ For time-coursed, non-real-time measurements, pulse metabolic labeling can be employed to look at proteoform turnover, as demonstrated with lipidated proteomes^175^ and specific lipidated proteins.^176^

## 7. Multiplexed detection of complex proteoforms

**Fig. 7:**
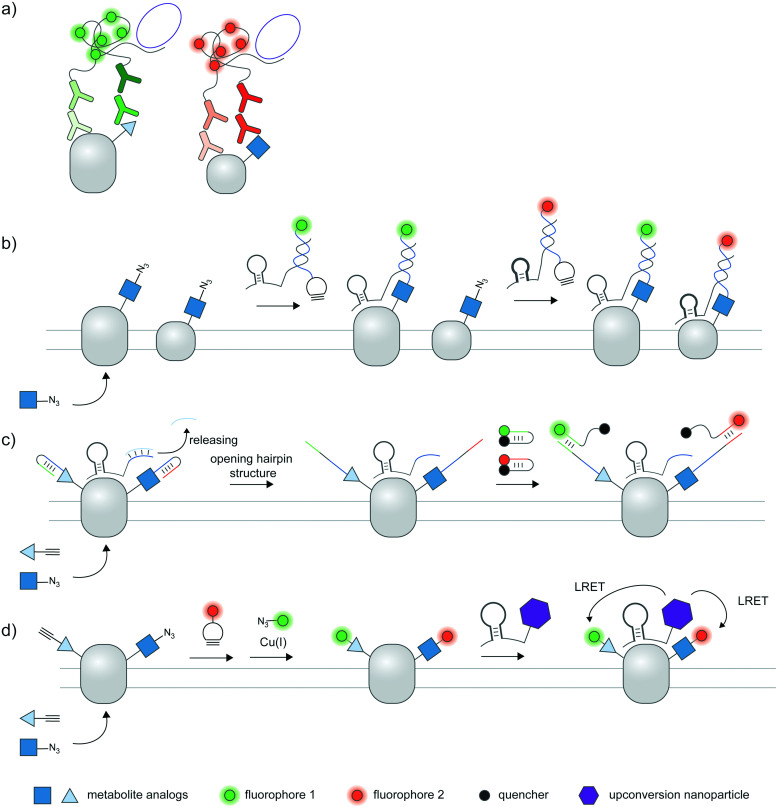
Multiplexed detection of proteoforms. (a) Multiplexed PLA as a basic strategy to detect multiple proteins or PTMs. (b) Detecting multiple specific proteins bearing the same PTM *via* multiple aptamers and metabolic labeling.^128^ (c) A hierarchical coding (HieCo)^180^ strategy to detect two PTMs on a specific protein. After metabolic labeling with two clickable sugar analogs, cascade & proximity-dependent hybridization events trigger binding of two molecular beacons in a protein- and sugar-specific manner, allowing simultaneous detection of two glycoforms. (d) Multiplexed energy transfer with an upconversion nanoparticle to detect two PTMs on a specific protein.^181^ The nanoparticle targeted to a specific protein serves as a sole LRET donor for two acceptor dyes, installed onto two sugar analogs *via* metabolic labeling.

Cellular proteoforms are often complex and can contain multiple PTMs;^177^ detecting these complex species remains an outstanding challenge. The use of two or more antibody-based reporters with different fluorophores have been employed for multiplexed detection of PTMs within single cells.^36,37,61^ Multiplexed live cell monitoring of proteoforms has also been reported using EGFP- and SNAP-tagged mintbodies.^178^ Multiplexed PLA could be coupled to protein- and PTM-specific antibodies to simultaneously visualize protein-specific PTMs and protein–protein interactions (Fig. 7a).^179^ Aptamer-assisted labeling can be used in combination with metabolic labeling to probe multiple proteins bearing the same PTM; heterodimerization of HER2–EGFR glycoforms, metabolically labeled with Ac_4_ManNAz, could be visualized using two aptamer–probe conjugates (Fig. 7b).^128^

Furthermore, several innovative strategies have been employed to tackle the challenge of multiplexed detection. To visualize glycoforms containing multiple types of monosaccharides at different compositions, Ac_4_ManNAz and Ac_4_FucAl probes were metabolically incorporated into the protein, followed by conjugation of two oligonucleotide probes, binding of an aptamer specific to MUC1 protein, and masking of the conjugated probes (Fig. 7c).^180^ Addition of unmasking oligonucleotides and molecular beacon probes allowed multiplexed detection of two sugars on the protein; different glycosylation patterns in different cell types can be monitored.

In another work, an upconversion nanoparticle was conjugated to an MUC1-specific aptamer.^181^ The nanoparticle provides two emission bands which allow luminescence resonance energy transfer (LRET) with two acceptor dyes—installed to two sugar types *via* metabolic labeling—upon a single near-infrared excitation (Fig. 7d). This system was successfully used for duplex imaging and quantification of fucosylated and sialylated MUC1, of which complex changes in glycosylation patterns can be tracked. The upconversion nanoparticle-conjugated aptamer was also used to identify phosphorylation and ubiquitination states of human epidermal growth factor receptor 2 (HER2).^182^ Here, ubiquitination was monitored through a cy5-labeled antibody for ubiquitin, while a small-molecule probe containing a Zn(ii)–cyclen moiety and cy3 detected phosphorylation on proteins.

The chemical binders based on binuclear zinc complexes for diphosphorylated Tau protein have also been reported with low affinity toward monophosphorylated or non-phosphorylated Tau.^183^ These reporters were used to visualize hyperphosphorylated Tau in hippocampal sections from Alzheimer's disease patients^183^ and in primary neuron culture from mouse.^184^

## 8. Potential platforms for high-throughput detection of proteoforms

**Fig. 8:**
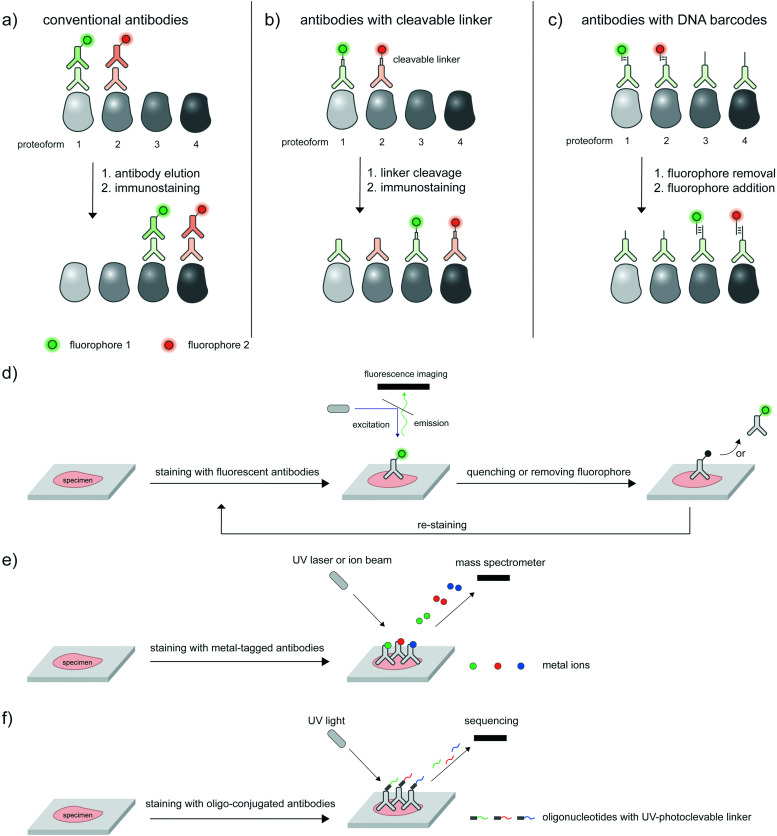
Potential technologies for high-throughput proteoform detection. These are multiplexed antibody-based protein profiling technologies currently used in spatial proteomic research. Iterative rounds of antibody staining can be accomplished *via*: (a) antibody stripping under optimized conditions, then restaining; (b) antibodies with cleavable fluorophores; and (c) DNA-barcoded antibodies as used in CODEX. (d) Iterative immunostaining and removal of labels/antibodies can be coupled to fluorescence microscopy to interrogate multiple proteoforms. (e) In lieu of fluorescence, mass cytometry-based techniques such as CyTOF can map released mass tags (rare-earth-metal isotopes) with high subcellular precision and multiplexity. (f) Digital spatial profiling sequences and quantifies indexed oligonucleotides photocleaved from a subcellular region of interest. In (e) and (f), antibodies are used to direct mass tags or indexed barcodes to desired biomolecules within the cell.

The ability to image proteoforms with highly complex modification states beyond two PTMs, or simultaneous imaging of multiple proteoforms, is difficult with current technologies. Tagging of genetically or metabolically incorporated probes often requires the use of mutually orthogonal bio-orthogonal chemistries,^185^ of which there are limited availabilities. While multiplexed amplification-based assays such as PLA are amenable to detecting up to 24 analytes,^186^ the extension of such assays to multicolor imaging in cells is limited by the color barrier of conventional fluorescence microscopy setup, which allows up to five spectrally separated fluorophores to be imaged at once. Modern microscopes with spectral unmixing and compensation capabilities,^187^ and ratiometric recoding of combinatorial fluorescent signals^188^ can overcome the color barrier, but are inevitably associated with higher errors from imperfect measurements. Instead of simultaneous imaging, iterations of immunostaining and signal removal can be performed with rounds of antibody stripping/elution and restaining^189^ (Fig. 8a), chemically cleavable fluorescent antibodies^190^ (Fig. 8b), and DNA-barcoded antibodies^191^ (Fig. 8c). Under optimized conditions such an iterative protocol (Fig. 8d) can be used to detect up to 40 protein targets, as demonstrated with iterative indirect immunofluorescence imaging (4i).^189^

Beyond direct imaging of fluorescently labeled proteins and PTMs, increasingly high-throughput platforms to interrogate spatial proteomics may be extended to multiplexed, high-throughput detection of proteoforms, provided that suitable proteoform labeling tools are in place. Such platforms include primer extension and imaging-based CODEX,^191^ mass cytometry-based CyTOF^192,193^ (Fig. 8e) and barcode sequencing-based Digital Spatial Profiling^194^ (Fig. 8f); Hickey *et al.*^195^ provides a state-of-the-art review on these multiplexed protein profiling technologies. Antibody-based reagents for the detection of proteins, PTM types, or site-specific PTMs are naturally applicable to these platforms, but proximity-based measurements/assays needed to establish the PTM/protein intramolecularity are currently not applicable. The challenge to create new proteoform labeling tools which provide compatible readouts with these high-throughput technologies represents exciting opportunities for chemical biology tool developers.

## 9. Conclusion and outlook

The chemical diversity and heterogeneity of cellular proteoforms give rise to their complex functions, but pose a grand challenge to their specific detection in the cellular context. Advances in protein and antibody engineering, protein and metabolic labeling, nucleic acid-based detection, and other innovative strategies have created molecular probes capable of highlighting spatial localization and temporal dynamics of these proteoforms. Despite their immense utility, these probes are to be used with caution and rigorous validations since they are limited in one characteristic or another, as outlined throughout the review. The field would benefit from continual refinement of probes targeting frequent, well-studied post-translational modification types, as well as creation of new tools for even more diverse classes of post-translational modifications and proteoforms. Linking molecular probes to instrumental platforms with spatially resolved, highly sensitive and multiplexed detection capability could enable broad dissection of functional roles of the most predominant proteoforms of cellular proteomes in the near future.

## Conflicts of interest

The authors declare no competing interests.

## Supplementary Material
